# HPV-relatedness definitions for classifying HPV-related oropharyngeal cancer patient do impact on TNM classification and patients’ survival

**DOI:** 10.1371/journal.pone.0194107

**Published:** 2018-04-17

**Authors:** Miren Taberna, Marisa Mena, Sara Tous, Miquel Angel Pavón, Marc Oliva, Xavier León, Jacinto Garcia, Marta Guix, Rafael Hijano, Teresa Bonfill, Antón Aguilà, Laia Alemany, Ricard Mesía

**Affiliations:** 1 Department of Medical Oncology, Catalan Institute of Oncology (ICO), ONCOBELL, IDIBELL, L’Hospitalet de Llobregat, Barcelona, Spain; 2 Cancer Epidemiology Research Program, Catalan Institute of Oncology (ICO), IDIBELL, L’Hospitalet de Llobregat, Barcelona, Spain; 3 University of Barcelona, Barcelona, Spain; 4 Centro de Investigación Biomédica en Red de Cáncer (CIBERONC), Madrid, Spain; 5 Centro de Investigación Biomédica en Red de Cáncer (CIBERESP), Madrid, Spain; 6 Department of Otorhinolaryngology, Hospital de Sant Pau, Barcelona, Spain; 7 Centro de Investigación Biomédica en Red de Bioingeniería, Biomateriales y Nanomedicina (CIBER-BBN), Madrid, Spain; 8 Cancer Research Program, IMIM, Hospital del Mar, Barcelona, Spain; 9 Department of Medical Oncology, Hospital del Mar, Barcelona, Spain; 10 Department of Otorhinolaryngology, Hospital del Mar, Barcelona, Spain; 11 Department of Medical Oncology, Hospital Universitari Parc Taulí, Sabadell, Barcelona, Spain; 12 Department of Otorhinolaryngology, Hospital Universitari Parc Taulí, Sabadell, Barcelona, Spain; 13 Department of Medical Oncology, Catalan Institute of Oncology (ICO), Badalona, Barcelona, Spain; Fondazione IRCCS Istituto Nazionale dei Tumori, ITALY

## Abstract

**Background:**

Given the different nature and better outcomes of oropharyngeal carcinoma (OPC) associated with human papillomavirus (HPV) infection, a novel clinical stage classification for HPV-related OPC has been accepted for the 8^th^ edition AJCC TNM (ICON-S model). However, it is still unclear the HPV-relatedness definition with best diagnostic accuracy and prognostic value.

**Material and methods:**

The aim of this study was to compare different staging system models proposed for HPV-related OPC patients: 7^th^ edition AJCC TNM, RPA stage with non-anatomic factors (Princess Margaret), RPA with N categories for nasopharyngeal cancer (MD-Anderson) and AHR-new (ICON-S), according to different HPV-relatedness definitions: HPV-DNA detection plus an additional positive marker (p16^INK4a^ or HPV-mRNA), p16^INK4a^ positivity alone or the combination of HPV-DNA/p16^INK4a^ positivity as diagnostic tests.

**Results:**

A total of 788 consecutive OPC cases diagnosed from 1991 to 2013 were considered eligible for the analysis. Of these samples, 66 (8.4%) were positive for HPV-DNA and (p16^INK4a^ or HPV-mRNA), 83 (10.5%) were p16^INK4a^ positive and 58 (7.4%) were double positive for HPV-DNA/p16^INK4a^. ICON-S model was the staging system, which performed better in our series when using at least two biomarkers to define HPV-causality. When the same analysis was performed considering only p16^INK4a^-positivity, RPA stage with non-anatomic factors (Princess Margaret) has the best classification based on AIC criteria.

**Conclusion:**

HPV-relatedness definition for classifying HPV-related OPC patient do impact on TNM classification and patients’ survival. Further studies assessing HPV-relatedness definitions are warranted to better classify HPV-related OPC patients in the era of de-escalation clinical trials.

## Introduction

Human Papillomavirus (HPV) related oropharyngeal squamous cell carcinoma (OPC) represents a distinct entity, with different clinical, epidemiological and molecular features, treatment responsiveness and survival [1]. A 58–74% reduction in the risk of death has been observed in HPV-related OPC when compared with HPV non-related OPSCC associated to the classical risk factors, tobacco and alcohol [2,3]. Taking into account these differences, it became evident that the 7^th^ edition American Joint Committee on Cancer (AJCC) TNM staging system does not reflect accurately patient survival in order to drive therapeutic decisions for HPV-related OPC patients. This disparity in prognosis affects clinical trials design, evaluation of treatment outcomes, research and communication between head and neck cancer health community. Therefore, several approaches for HPV-OPC new staging systems have already been proposed and one of them has been selected for the 8^th^ edition AJCC TNM.

Importantly, OPC HPV relatedness definition is still under development and the new staging system proposals have not adhered to a strict definition of viral aetiology of OPC cases. It is already well understood that HPV-DNA detection alone is not sufficient to classify an OPC as HPV-related since the presence of HPV-DNA could only reflect a transient infection [4]. Additionally, the detection of p16^INK4a^ expression is not specific for HPV activity [4,5]. The choice of HPV-relatedness definition do impact on patients survival; p16^INK4a^ high expression but HPV-DNA-negative OPC patients have showed significantly less favourable survival than patients with p16^INK4a^ high expression and HPV-DNA-positive tumours [6].

Before the new TNM edition was accepted, several proposals were described. Huang and colleagues [7] developed a new staging system by using a recursive partitioning analysis (RPA) and a adjusted hazard ratio (AHR) model for overall survival (OS), comprising both anatomic (TNM) and non-anatomic parameters such as age and tobacco. Dahlstrom and colleagues [8] were unable to validate all the categories of this proposal so they created a new staging system by incorporating traditional N stage categories used for nasopharyngeal cancer [8,9]. Finally, O’Sullivan and colleagues, with The International Collaboration on Oropharyngeal cancer Network for Staging (ICON-S) [10], validated in a new cohort the previous described RPA and AHR systems, and finally proposed an AHR-New model for the 8^th^ edition of the TNM classification, which was finally accepted. Table 1 summarized the 7^th^ edition AJCC TNM staging system, and the three main new staging systems proposals: RPA stage with non-anatomic factors (Princess Margaret) [7], RPA with N categories for nasopharyngeal cancer (NPC) (MD-Anderson) [8] and AHR-new (ICON-S) [10]. Importantly, none of them used a uniform HPV testing method to define HPV-relatedness (Table 1).

**Table 1:** Summary of 7^th^ edition AJCC TNM and new staging system proposals.

Staging System	*N*	HPV-relatedness definition	New proposal	Stage Classifications	5 years OS by Stage
**7**^**th**^ **edition AJCC TNM** [11]	NA	NA	NA	Stage I (T1N0)	NA
				Stage II (T2N0)	
				Stage III (T3N0 or T1-T3N1)	
				Stage IVa (T4aN0-1 or T1-T4aN2)	
				Stage IVb (T4b or T1-T4bN3)	
**Princess Margaret** Huang et al. [7]	573	p16^INK4a^ IHC	RPA stage with non-anatomic factors	RPA-I (T1-3N0-N2c ≤ 20 PY)	RPA-I: 89%
				RPA-II (T1-3N0-N2c >20 PY)	RPA-II: 64%
				RPA-III (T4 or N3_age ≤ 70)	RPA-III: 57%
				RPA-IVA (T4 or N3_age >70)	RPA-IVA: 40%
**MD Anderson** Dahlstrom et al. [8]	661	HPV-DNA ISH or p16^INK4a^ IHC or both	RPA with N categories for NPC	Stage IA (T1N0-N2)	IA: 94%
				Stage IB (T2N0-N2)	IB: 87%
				Stage II (T1-T2N3 or T3N0-N3)	II: 76%
				Stage III (T4)	III: 69%
**ICON-S** O’Sullivan et al. [11]	1907	HPV-DNA ISH or p16^INK4a^ IHC	AHR-New (ICON-S)	Stage I (T1-T2N0-N1)	I: 88%
				Stage II (T1-T2N2 or T3N0-N2)	II: 81%
				Stage III (T4 or N3)	III: 65%

NA: Not applicable; IHC: immunohistochemistry; ISH: in situ hybridization; OS: Overall survival; RPA: recursive partitioning analysis; PY: Pack-years; NPC: nasopharyngeal cancer; ICON-S: The International Collaboration on Oropharyngeal Cancer Network for Staging; AHR: adjusted hazard ratios.

A novel clinical stage classification for HPV-related OPC has already been described for the 8^th^ edition AJCC TNM, based on the ICON-S proposal [11] Main differences among 7^th^ and 8^th^ TNM editions including HPV-related oropharyngeal cancer patients are summaryzed on Table 2.

**Table 2:** Main differences among 7^th^ and 8^th^ TNM editions including HPV-related oropharyngeal cancer patients. Modified from Taberna et al. Annals of Oncology 2017 [1].

Characteristics	7^th^ Edition TNM	8^th^ Edition TNM ICON-S
**Stage Classifications**	-Stage I (T1N0)	-Stage I (T1-T2N0-N1)
	-Stage II (T2N0)	-Stage II (T1-T2N2 or T3N0-N2)
	-Stage III (T3N0 or T1-T3N1)	-Stage III (T4 or N3)
	-Stage IVa (T4aN0-1 or T1-T4aN2)	-Stage IV (M1)
	-Stage IVb (T4b or T1-T4bN3)	
	-Stage IVc (M1)	
**Main N (lymph node) differences**	-N1: metastasis in a single ipsilateral lymph nodes, < 3 cm	-N1: ipsilateral metastasis in lymph node(s), < 6 cm
	-N2a: metastasis in a single ipsilateral lymph node > 3 cm but < 6 cm.	-N2: bilateral or contralateral metastasis in lymph node(s), < 6 cm*
	-N2b: metastasis in multiple ipsilateral lymph nodes, < 6 cm	
	-N2c: metastasis in bilateral o contralateral lymph nodes, < 6 cm	
**Main T (tumor) differences**	T4a: Tumor invades the larynx, extrinsic muscle of tongue, medial pterygoid, hard palate or mandible	T4: Tumor invades any of the following: larynx, deep/extrinsic muscle of tongue, medial pterygoid, hard palate, mandible, lateral pterygoid muscle, pterygoid plates, lateral nasopharynx, skull base or encases carotid artery **
	T4b: Tumor invades lateral pterygoid muscle, pterygoid plates, lateral nasopharynx, skull base or encases carotid artery	

HPV: Human papillomavirus; OPSCC: Oropharyngeal squamous cell carcinoma; NA: Not applicable; OS: Overall survival; ICON-S: The International Collaboration on Oropharyngeal cancer Network for Staging; T: tumor; N: lymph node; M: metastasis

* Because 5-years OS was similar among N1, N2a and N2b, they re-termed the N categories.

** Because 5-years OS was similar among T4a and T4b, they were no longer subdivided and it was re-termed as T4.

To better understand and compare the different staging systems proposed and the accepted one, we have validated them in an independent data set, with different HPV-relatedness definitions.

## Materials and methods

### Study population and design

We carried out a retrospective study to assess the prognostic and predictive value of HPV viral DNA and of HPV-related carcinogenic biomarkers, in formalin-fixed paraffin-embedded (FFPE) samples of OPC, consecutively selected from four different hospitals from Catalonia (Catalan Institute of Oncology-ICO-Hospital Universitari de Bellvitge; Hospital de Sant Pau, Hospital del Mar and Hospital Parc Taulí) [12] from 1991 to 2013. Nested within this study was this sub-analysis to compare the different staging systems proposed for HPV-related OPC patients in an independent data set with different HPV-relatedness definitions. Cases were pathologically confirmed and metastatic patients discarded. Demographic and clinical information was extracted from clinical reports of each center and all data was fully anonymized. All methods were carried out in accordance with relevant guidelines and regulations. The protocol was approved by the Institutional Review Broad of each participating hospital, which required no informed consent to use archived tumor samples and retrospective data.

### Histopathological evaluation and laboratory analysis

All determinations were centrally performed in FFPE at the Catalan Institute of Oncology, the detailed methods used for immunohistochemistry (IHC), HPV-DNA detection, genotyping, and HPV E6*I mRNA performance have been reported elsewhere [13]. Hematoxylin and eosin stained slides were used to confirm presence and estimate the proportion of invasive SCC in the specimen as well as to classify histopathological features. Briefly, we used Roche mtm Laboratories AG IHC (Heidelberg) for p16^INK4a^ determination and SPF-10 polymerase chain reaction (PCR) and a DNA enzyme immunoassay (DEIA) to test for the presence of HPV-DNA in all cases. Genotyping was performed using reverse hybridization line probe assay (LiPA25_v1). All samples testing positive for HPV-DNA underwent E6*I mRNA detection at DKFZ, Heidelberg, Germany, as developed by Halec and colleagues [14]. p16^INK4a^ IHC was considered positive when the pattern showed a strong and diffuse nuclear and cytoplasmic staining in at least 70% of the tumor [15].

We used different definitions for HPV-positivity to evaluate the staging system proposals. Firstly, cases were stratified by tumor HPV status and were considered HPV-related if HPV-DNA PCR and (p16^INK4a^ IHC or HPV-mRNA PCR) determination were positive. Secondly, cases were analyzed by p16^INK4a^ IHC expression alone, as p16^INK4a^ is widely used in clinical settings and some of the staging system proposals evaluated in this study use only this biomarker to define HPV-positivity. Finally, cases were defined as HPV-positive when both HPV-DNA and p16^INK4a^ expression remained positive, as this combination has been shown to have highest specificity to describe HPV-transformed OPCs [16] and its implementation in the clinical setting is easier compared to mRNA HPV detection.

### Statistical considerations

We estimated the rates of OS by means of the Kaplan–Meier method. Due to the low number of cases, Nelson-Aalen estimates of the OS were also performed without observing statistically significant differences between the two methods. We used log-rank test to evaluate the equality of survivor functions across two or more groups and a univariate Cox model (proportional hazard model) was also performed for each stage classification. Trend test was used to evaluate the trend of the survival function across the three or more ordered groups. The comparison of these models was done using AIC (Akaike Information Criterion) which estimate the quality of each model, relative to each of the other models; these criteria penalize the number of parameters in the model selecting the one with the lowest AIC as the best model.

## Results

A total of 788 consecutive OPC cases diagnosed from 1991 to 2013 were obtained and considered eligible for the analysis. Of these samples, 66 (8.4%) were HPV-related (HPV-DNA PCR and (p16^INK4a^ IHC or HPV-mRNA PCR) positive) and had a non-metastatic stage. Of note, all samples double positive for HPV DNA and p16 ^INK4a^ IHC were also positive for HPV-mRNA. The demographic and clinical characteristics of the 66 HPV-related non-metastatic OPC evaluable cases are shown in Table 3. HPV-related OPC patients had a mean age of 60.2 (SD: 13.8), 45 were male (68.2%), 34 were smokers (51.5%); 55 were diagnosed during the period of 2000–2013 (83.3%) and the tonsil was the most common subsite (45, 68.2%). The majority of patients (48, 72.7%) were treated with chemo(radiotherapy) and only 16 (24.2%) underwent a primary surgery procedure. After a median follow-up of 5.0 years (Range: 0.2–22.7), 44 (66.7%) patients were still alive.

**Table 3:** Demographic and clinical characteristics of the 66 HPV-related non-metastatic OPC included on the analysis.

Characteristic	HPV/DNA+ AND (HPV-mRNA+ OR p16^INK4a^+) (N = 66)	
	*N*	%
**Age:** Mean (SD)	60.2 (13.8)	NA
**Gender**		
Male	45	68.2
Female	21	31.8
**Period of diagnosis**		
1991–1999	11	16.7
2000–2009	36	54.5
2010–2013	19	28.8
**Smoking status**		
Non Smoker	32	48.5
≤20 PY	14	21.2
>20 PY	20	30.3
**Alcohol status**		
Non Drinker	36	54.5
Moderate (≤100 g/d)	24	36.4
Heavy (> 100g/d)	20	9.1
**Subsite**		
Tonsil	45	68.2
Base of the tongue	13	19.7
Other	8	12.1
**Treatment**		
Palliative	2	3.0
Surgery	16	24.2
RT/CT	48	72.7

HPV: Human papillomavirus; OPC: Oropharyngeal squamous cell carcinoma; SD: Standard deviation; NA: not applicable; PY: pack-year; g/d: grams per day; RT: Radiotherapy; CT: Chemotherapy.

**Fig 1:**
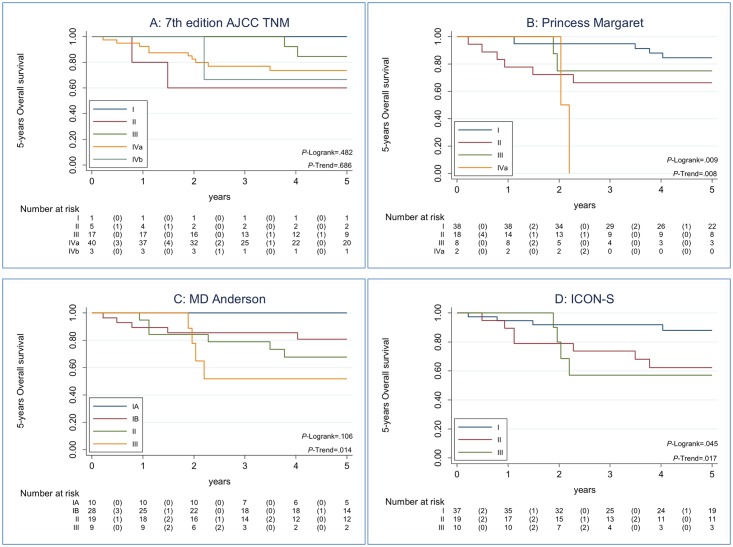
Kaplan-Maier estimates of Overall Survival among the 66 HPV-related OPSCC patients, according to each staging system. Data on 5-years Overall Survival and Trend test are shown according to each staging system for HPV-related OPSCC patient. Panel A showed Kaplan-Meier curve for the 7^th^ edition AJCC TNM classification with a non-statistically significant Trend test P-value. Panel B showed Kaplan-Meier curve for RPA stage with non-anatomic factors (Princess Margaret). Panel C showed Kaplan-Meier curve for RPA with N categories for nasopharyngeal cancer (MD-Anderson). Panel D showed Kaplan-Meier curve for AHR-new (ICON-S). For Panel B, C and D Trend tests were statistically significant, indicating that the trend of the survival function across the three or more stages classifies them in a linear tendency. Panel D, AHR-new (ICON-S), has the best classification based on AIC criteria.

We performed the comparison among the 7^th^ AJCC TNM edition and the different staging systems proposed for HPV-related OPC patients based on three different HPV-relatedness definitions. Fig 1 shows the Kaplan-Meier estimates of 5-years OS and the Trend test resulted according to each staging system, for HPV-DNA PCR and (p16^INK4a^ IHC or HPV-mRNA PCR) positive cases. Following the AIC criteria for models comparison, we observed that all the staging classifications improved the OS assessment in HPV-related OPC patients compared with the 7^th^ edition AJCC TNM classification (Table 4). *P*-trend test was statistically significant for all the new proposal staging systems and non-significant for the 7th edition AJCC TNM staging system (*P* = .685). Based in our series, the best classification was AHR-new (ICON-S) (*P* = .05; AIC: 117.5; Trend test *P* = .02), followed by RPA with N categories for NPC (MD-Anderson) and RPA stage with non-anatomic factors (smoking and age) (Princess Margaret).

**Table 4:** HPV-related OPC patients classify by AIC criteria and Trend tests results according to each HPV-relatedness definition.

**HPV-DNA and (p16**^**INK4a**^ **or HPV-mRNA) positive patients (*N* = 66)**				
**TNM classification**	**Log-likelihood ratio test *P*-value**	**Trend test *P*-value**	**AIC**	**Conclusion**
7th edition AJCC TNM	0.4655	0.6859	124.08	Worse than the null model
Princess Margaret	0.0535	0.0081	118.00	
MD Anderson	0.0510	0.0144	117.89	
**ICON-S**	0.0467	0.0166	117.53	**Best AIC model**
Null model			119.66	
**p16**^**INK4a**^**-positive patients (*N = 83*)**				
**TNM classification**	**Log-likelihood ratio test *P*-value**	**Trend test *P*-value**	**AIC**	**Conclusion**
7th edition AJCC TNM	0.7919	0.4243	221.29	Worse than the null model
**Princess Margaret**	0.0222	0.0121	202.56	**Best AIC model**
MD Anderson	0.2102	0.0615	216.46	Worse than the null model
ICON-S	0.0211	0.0207	211.27	
Null model			214.99	
**HPV-DNA and p16**^**INK4a**^ **positive patients (*N = 58*)**				
**TNM classification**	**Log-likelihood ratio test *P*-value**	**Trend test *P*-value**	**AIC**	**Conclusion**
7th edition AJCC TNM	0.6801	0.2096	83.01	Worse than the null model
Princess Margaret	0.4171	0.2041	80.47	Worse than the null model
MD Anderson	0.1280	0.0427	77.62	Worse than the null model
**ICON-S**	0.0393	0.0371	74.84	**Best AIC model**
Null model			77.31	

HPV: Human papillomavirus; OPC: Oropharyngeal squamous cell carcinoma; AIC: Akaike Information Criterion; ICON-S: International collaboration on oropharyngeal cancer network for staging

**Fig 2:**
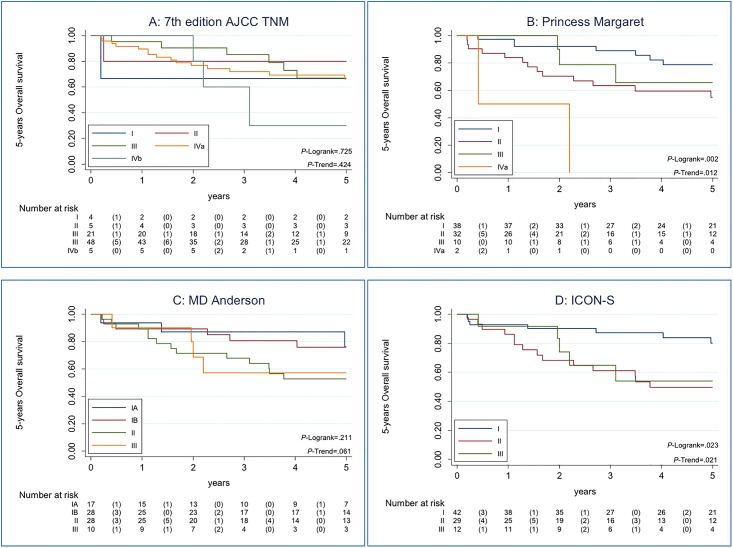
Kaplan-Maier estimates of Overall Survival among the 83 p16^INK4a^-positive patients, according to each staging system. Data on 5-years Overall Survival and Trend test are shown according to each staging system for HPV-related OPSCC patient. Panel A showed Kaplan-Meier curve for the 7^th^ edition AJCC TNM classification with a non-statistically significant Trend test p-value. Panel B showed Kaplan-Meier curve for RPA stage with non-anatomic factors (Princess Margaret). Panel C showed Kaplan-Meier curve for RPA with N categories for nasopharyngeal cancer (MD-Anderson). Panel D showed Kaplan-Meier curve for AHR-new (ICON-S). For Panel B and D Trend tests were statistically significant, indicating that the trend of the survival function across the three or more stages classifies them in a linear tendency. Panel B, RPA stage with non-anatomic factors (Princess Margaret), has the best classification based on AIC criteria.

The same analysis was performed taking into account only p16^INK4a^-positivity (*N* = 83), as it is the regular technique use in the clinical setting. Fig 2 shows the Kaplan-Meier estimates of 5-years OS and the Trend test result according to each staging system. Differently from the previous analysis, RPA stage with non-anatomic factors (Princess Margaret) has the best classification based on AIC criteria (*P* = .02; AIC: 202.6; Trend test *P* = .01), followed by AHR-new (ICON-s) and RPA with N categories for NPC (MD-Anderson).

**Fig 3:**
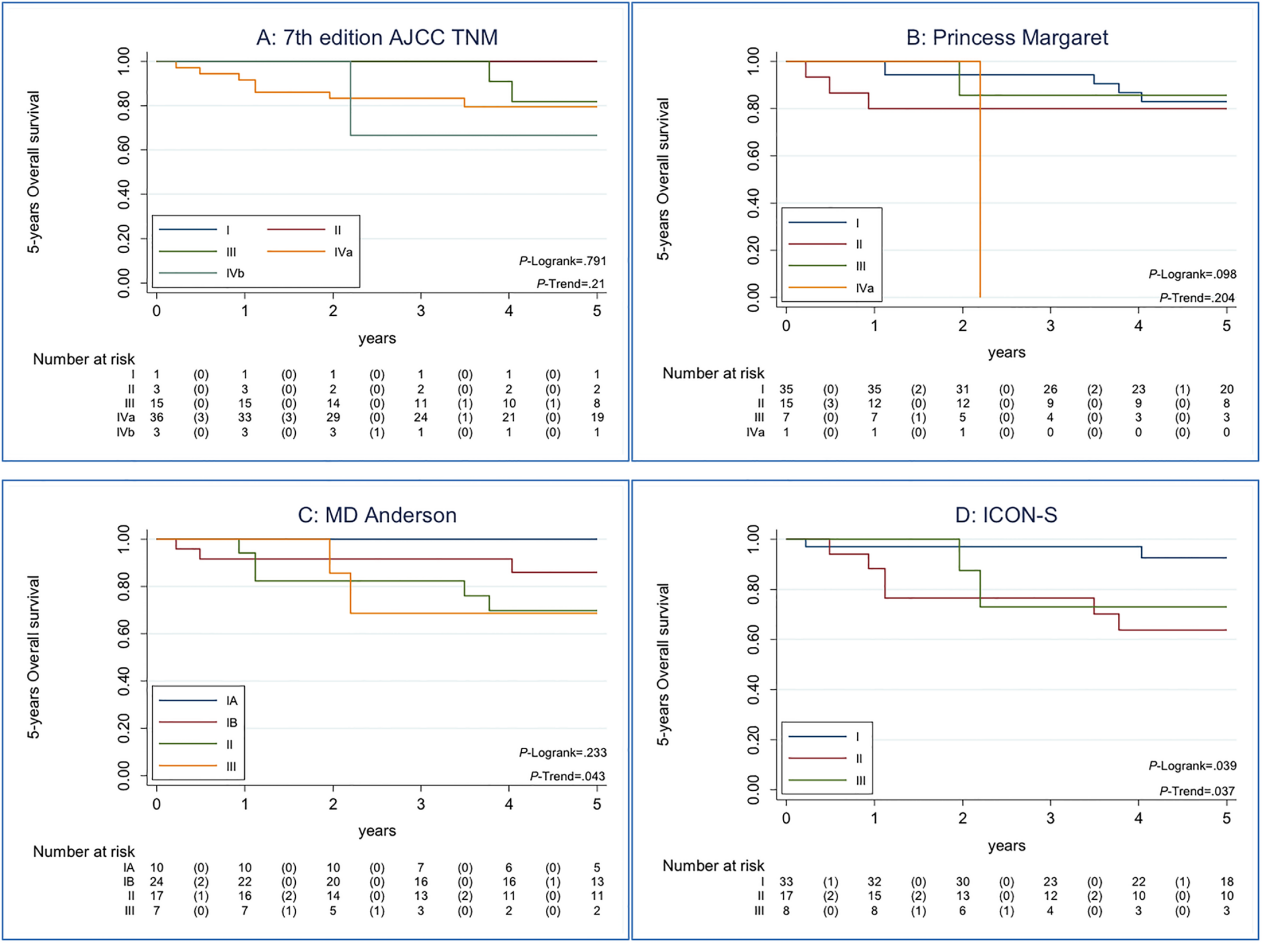
Kaplan-Maier estimates of Overall Survival among the 58 HPV-DNA/p16^INK4a^ double positive patients, according to each staging system. Data on 5-years Overall Survival and Trend test are shown according to each staging system for HPV-related OPSCC patient. Panel A showed Kaplan-Meier curve for the 7^th^ edition AJCC TNM classification with a non-statistically significant Trend test p-value. Panel B showed Kaplan-Meier curve for RPA stage with non-anatomic factors (Princess Margaret). Panel C showed Kaplan-Meier curve for RPA with N categories for nasopharyngeal cancer (MD-Anderson). Panel D showed Kaplan-Meier curve for AHR-new (ICON-S). For Panel C and D Trend tests were statistically significant, indicating that the trend of the survival function across the three or more stages classifies them in a linear tendency. Panel D, AHR-new (ICON-S), has the best classification based on AIC criteria.

Finally, we performed the analysis using the combination of HPV-DNA and p16^INK4a^ positivity (*N* = 58). Kaplan-Meier estimates of 5-years OS and the Trend test are represented on Fig 3. The best classification taking into account the AIC model was again the AHR-new (ICON-S) (*P* = .04; AIC: 74.8; Trend test *P* = .04), followed by RPA with N categories for NPC (MD-Anderson).

Results from HPV-related OPC patients’, p16^INK4a^-positive patients’ and the double positivity for HPV-DNA/p16^INK4a^ patients’ analysis are summarized on Table 4.

## Discussion

To our knowledge, this is the first attempt to validate the different staging systems proposed for HPV-related OPC patients, in an independent data set using different HPV-relatedness definition to determine HPV-causality.

We observed that all the staging classifications proposed improve the overall survival assessment in HPV-related OPC patients compared with the current 7^th^ edition AJCC TNM classification independent of the HPV-relatedness definition. ICON-S model proposed by O’Sullivan and colleagues has been accepted for the 8^th^ edition AJCC TNM [10]. In accordance with it, ICON-S model was the staging system which performed better in our series when using at least two biomarkers to define HPV-causality (HPV-DNA and (p16^INK4a^ or HPV-mRNA) or double positivity for HPV-DNA/p16^INK4a^). Nevertheless, this result was not reached when HPV-positive patients were defined based on p16^INK4a^ expression alone. In this scenario, Princess Margaret model was the sating system which performed better following the AIC criteria. This finding, suggest that HPV-relatedness definitions for classifying HPV-related OPC patients do impact in OS and TNM classifications. It is important to mention that none of the three new staging system evaluated used in their criteria an uniform test with at least two biomarkers (*i*.*e*. p16^INK4a^/HPV-DNA positivity) to define an HPV-related OPC patient. As we have demonstrated in a previous publication carried out by our group [13], using either or both E6*I mRNA or p16^INK4a^ in addition to HPV-DNA is a good combination for HPV-causality detection in OPC, as detection of HPV-DNA alone is not sufficient to establish causality and using p16^INK4a^ IHC alone is questionable, because a subset of HPV-DNA and mRNA negative OPCs show diffuse p16^INK4a^ staining, indicating expression is not specific for HPV activity and maybe over-expressed by another cause (for example, a Rb mutation). The discordant rates are around 20% of the cases [17], being mostly p16^INK4a^-positive and HPV-DNA-negative cases. On the other hand, HPV-mRNA detection is difficult to reproduce on the clinical setting, therefore the combination of p16^INK4a^ IHC and HPV-DNA detection (by PCR or ISH) has been studied. A recent meta-analysis showed that this combination is the method with the highest accuracy to diagnose HPV-related OPCs [16]. Importantly, Rietbergen et colleagues demonstrated, that patients with p16^INK4a^-positive but HPV-DNA-negative OPC showed a significantly less favourable survival than patients with p16^INK4a^-positive and HPV-DNA-positive tumors (P <0.001) [6]. We have recently validated these data on an independent series [12], indicating that p16^INK4a^ expression alone is not an appropriate diagnosis and prognosis biomarker for an accurate HPV-relatedness definition. In line with our results, data from a recent report by Nauta and colleagues evaluating the 8^th^ TNM classification on p16 ^INK4a^-positive OPC in Netherlands, highlight the importance to perform additional HPV DNA-testing when predicting OPC patients prognosis [18]. Moreover, Boscolo-Rizzo and colleagues have also defended the use of more accurate biomarkers beside p16^INK4a^ expression alone to classify HPV-related OPC patients [19]. As these authors explained in a recent article, the attributable fraction of HPV-related OPC variate geographically, assuming that p16^INK4a^ sensitivity and specificity are the same in all regions (high versus low attributable fractions), its diagnostic positive predictive value will drop considerably if the *a priori* probability of having a HPV-positive OPSCC is lowered [19]. This information is extremely important in order to classify accurately HPV-related OPC patients within the TNM staging system. In an era where de-escalation clinical trials evaluating surgical and conservative treatments are under development, an OPC patient’ misclassification could seriously affect their quality of life and survival.

Noteworthy, recent results from the ECOG-1308, the first de-escalation clinical trial for HPV-related OPC published [20] have demonstrated that clinical complete response to induction chemotherapy could select patients for reduced-dose IMRT (54 Gy) in combination with cetuximab with 2-years progression free survival (PFS) of 80% and significant improved swallowing and nutritional status. Importantly in this study, all treatment failures were among patients with a >10 PY smoking history, and in a post hoc analysis 2-years PFS was significantly higher among patients with ≤ 10 PY compared with dose with >10 PY (92% v %7%; *P* = .0014). Importantly, tobacco use seems to be diverse in North America with respect to Europe. Therefore, different risk factor exposure may contribute to a combined risk situations [19] according to the intermediate and high risk profile defined as previously by Ang and colleagues [2]. Despite ICON-S model was the staging system which performed better in our series, adding non-anatomic factors for the TNM staging system should be further considered, as it has been suggested by other groups before [9].

The strength of the present study is to evaluate the different staging systems proposed for HPV-related OPC patients, in an independent data set with different HPV-relatedness definitions. Nevertheless, the most important limitation is the low rate of HPV-positive OPC patients included in the analysis, since HPV-related OPC attributable fraction in our country is still low in comparison with other geographic regions like US or North Europe.

A novel clinical stage classification for HPV-related OPC has already been described for the 8^th^ edition AJCC TNM. Nevertheless, further studies about HPV-relatedness definitions are warranted in larger series of cases to better classify HPV-related OPC patients in an era of de-escalation clinical trials.

## Supporting information

S1 File Agregated_Data. (XLSX)
